# An exploration of Ubuntu leadership using interactive qualitative analysis

**DOI:** 10.3389/fpsyg.2025.1686493

**Published:** 2025-11-28

**Authors:** Anton Grobler, Kerryn Powell

**Affiliations:** Graduate School of Business Leadership, University of South Africa, Pretoria, South Africa

**Keywords:** Ubuntu leadership, Afrocentric leadership, organizational contexts, Southern Africa, interactive qualitative analysis

## Abstract

**Introduction:**

Many scholars have attempted to conceptualize *Ubuntu* leadership, as a specific expression of Afrocentric leadership, mainly from a philosophical perspective, using existing literature, and within the broader society and not within organizations. This study explores the nature of *Ubuntu* leadership in an organizational context, from a pure contextual perspective.

**Methods:**

The methodology used is the Interactive Qualitative Analysis, which is regarded to be a participatory technique, focused on the co-creation of knowledge through participation, an important element of *Ubuntu*. Through purposive sampling, data was collected from three focus groups of organizational leaders who met IQAs criteria of “distance and power over” the phenomenon.

**Results:**

The findings identified affinity groupings across the three System Influence Diagrams, mapping out the complex interconnections, indicating how *Ubuntu* values interact in leadership practice. The three System Influence Diagrams were intuitively integrated, resulting in *Transformational focus and ability* and *Authenticity, ethics and accountability* as primary and secondary drivers respectively, *Team synergy* as pivot, and *Harmonious inclusive leadership and culture* as well as *Individual, team and organizational growth and success* as outcomes (secondary and primary respectively).

**Discussion:**

*Ubuntu* leadership is framed as being transformational, inclusive, and ethical and centers around people and relationships. This study provides a context sensitive conceptualization of *Ubuntu* leadership, recommended for use by organizations, educators, and scholars to foster *Ubuntu* leadership in practice, promoting success and growth at individual, team, and organizational levels. Recommendations for future research were formulated, addressing the limitations of the present study.

## 1 Introduction

African organizations should leverage their cultural heritage to compete in the global marketplace by embracing uniquely African management principles grounded in the philosophy of *Ubuntu* (Mbigi, 1997; Sibanda and Grobler, 2023). *Ubuntu* is considered to be a unique Afrocentric approach to leading and managing by focusing on people and their dignity (Bolden and Kirk, 2009; Walumbwa et al., 2011; Grobler and Singh, 2018; Mangaroo-Pillay, 2025; Nelwamondo and Price, 2025; Nwozaku, 2023; Van Norren and Beehner, 2021; Zondo, 2022). Leadership in general is mostly conceptualized as a Western epistemological construct, typically rooted in individualism, hierarchy, rationality, and goal-oriented efficiency. This is in contrast with *Ubuntu*, grounded in communalism, relationality, interconnectedness, and shared humanity. This presents profound conceptual, practical, and ethical challenges, and it is therefore important to study *Ubuntu* leadership, through a contextual lens, and specifically within an organizational context.

*Ubuntu* leadership is a specific expression of Afrocentric leadership (Grobler and Singh, 2018; Nussbaum, 2003), with the latter being important to drive social changes through its emotive and inclusive nature, necessary for the creation of a promising, positive future. This positive future is built on the foundation of humanistic principles which value individual differences, authenticity and serving the community (Mangaliso et al., 2021; Sibanda and Grobler, 2023; Osa, 2019; Zondo, 2022). Lovemore Mbigi is a frequently cited scholar in African leadership literature, and Mangaliso (2001) and Mangaliso et al. (2021) argues that people are at the heart of African culture, and crafting *Ubuntu* into organizational leadership can positively affect an organization's performance, success and sustainability (Mbigi, 2007; Mangaroo-Pillay, 2025).

Many of the scholars focused on *Ubuntu* in the broader society and from a philosophical perspective. Valuable contributions have been made by (just to name a few), Mbigi (1997), Mangaliso (2001), Pérezts et al. (2020) and Zondo (2022). Brubaker (2013) Evans et al. (2021); Laloo (2022); Mangaliso et al. (2021), Molose et al. (2019) and Tauetsile (2021) contend however that there is a need for further discussion on the precise nature of *Ubuntu* within organizations and whether it can be conceptualized as a distinct model of leader behavior. Mangaliso et al. (2021, 130) is further of the opinion that “practices of *Ubuntu* with regard to humanity, care, sharing, teamwork spirit, compassion, dignity, consensus decision-making systems, and respect for the environment are all positive elements that could make a contribution toward the improvement of corporate performance”.

The *Ubuntu* philosophy is widely embraced by Nguni-speaking communities in southern Africa, with equivalent expressions found beyond South Africa in other sub-Saharan African countries. Broodryk (2005) notes that the concept of *Ubuntu* is articulated in various African languages, consistently highlighting its foundation in collectivism and communal relationships. Consequently, Southern Africa will be used for the geographical demarcation of this study.

Prior to this study, the authors conducted a systematic literature review of published, peer-reviewed theoretical and empirical studies within an organizational context over a 25 year period, investigating how *Ubuntu* leadership has been portrayed in organizational contexts. The review revealed four key themes: (i) *Ubuntu* within organizational contexts is regarded mostly as a relational concept; (ii) There is agreement that *Ubuntu* could be conceptualized as a leadership or management style; (iii) *Ubuntu*-related leadership can be described as participatory and values based; (iv) Researchers are calling for blended leadership approaches in Africa. This study empirically examines all four themes, either directly or indirectly, adopting a contextual explorative process.

### 1.1 Purpose and objectives

The purpose of this study is to provide a conceptualization of *Ubuntu* leadership within an organizational context using the Interactive Qualitative Analysis (IQA) technique, which supports the contextual and community-driven nature of the subject of investigation. The objectives are firstly to identify and define the components of *Ubuntu* leadership, and secondly, to explain the interconnectedness of the elements (possible causes or drivers and effects or outcomes), through the mapping of causal relationships in a visual form, showing how different themes interact. This study and methodology will be done through consultation and consensus of the participants” lived experiences, making it a truly insider and thus context sensitive conceptualization of *Ubuntu* leadership.

The uniqueness of this study is fourfold (i) it is based on the qualitative exploration of the concept (using IQA), which is different from the various (valuable) philosophical, literature, artificial intelligence based studies (defining it from an etic perspective), (ii) it focuses on *Ubuntu* leadership within an organizational context (the majority of studies mainly looked at *Ubuntu* leadership in the broader society), (iii) it goes beyond the definition of *Ubuntu* leadership (which is multi-facetted) as it investigates the interactions between the elements it comprises of, and (iv) it includes leaders from a diverse range of organizations and is not confined to a particular industry or entity, thereby increasing its comprehensiveness; interpretability and transferability.

### 1.2 *Ubuntu* and *Ubuntu* leadership: a concise literature review

*Ubuntu* is characterized as a communal philosophy emphasizing values such as survival, solidarity, compassion, respect, and dignity (Brubaker, 2013; Mbigi, 2007; Ntibagirwa, 2018; Nwozaku, 2023; Osa, 2019; Yawson, 2017; Zondo, 2022). It is encapsulated in the isiXhosa proverb “umntu ngumntu ngabantu,” meaning a person is a person through others (Akpey-Mensah and Muchie, 2019; Asamoah and Yeboah-Assiamah, 2019; Mangaliso et al., 2021). Literature often describes *Ubuntu* as humaneness, embodying a spirit of caring, community, harmony, and mutual respect (Mangaliso, 2001; Mangaroo-Pillay, 2025; Zondo, 2022). (Broodryk 2006a,b) suggests that values of caring, sharing, and compassion are essential for assessing humane presence within organizations.

In cross-cultural leadership literature, *Ubuntu* leadership is seen as humane-oriented, values-based, collectivist, and focused on collective wellbeing, thus inherently people-centered emphasizing respect and inclusion of all stakeholders. This leadership style prioritizes collective interests over individual ones (Grobler and Singh, 2018; Grobler and Koen, 2024; Nelwamondo and Price, 2025). A review by Wanasika et al. (2011) utilized qualitative and quantitative analyses of data from the GLOBE project, revealing high levels of group solidarity and humane-oriented leadership.

Khoza (2012) introduces the concept of attuned leadership, rooted in African humanism, which encompasses self-attunement, ethical awareness, and historical consciousness. While existing studies provide valuable insights into *Ubuntu* leadership, they should be complemented by local studies reflecting the lived experiences of southern African leaders. For instance, Molose et al. (2019) conducted a study using interviews to develop an *Ubuntu* instrument for the hospitality industry, focusing on dimensions of survival, respect, solidarity, compassion, and collectivism.

Given its relational nature, *Ubuntu* leadership is closely linked to ethical leadership theory (Nicolaides and Duho, 2019; Sipondo, 2025). Metz (2007, 2011) proposes a moral theory based on *Ubuntu* that emphasizes harmony and community development. However, there are calls to consider both positive and negative aspects of *Ubuntu*, including potential exclusivity and tribalism (Booysen, 2016; Malunga, 2006). Concerns have been raised about the exclusionary nature of *Ubuntu* leadership by several authors (Wanasika et al., 2011; Dorfman et al., 2012; Kamoche, 2011; Nicolaides and Duho, 2019).

Core Africanist Ubuntu scholarship reinforces these perspectives. Ubuntu is regarded as empowering people to love and respect each other (Teffo, 1999). In addition, it is seen as the root of African philosophy where ethical humanity is recognizing the humanity of others and establishing just and caring dependency relationships with them, which applies to all indigenous people of sub-Saharan Africa as well as the rest of the world (Ramose, 1999). Furthermore, Mugumbate and Chereni (2020, 6) describe Ubuntu as values and practices and the nuances thereof, as what makes people of African origin authentic human beings and seen as part of a larger and more significant relational, communal, societal, environmental and spiritual world. Samkange and Samkange (2013, 458) state that Ubuntu is an African philosophy, which literally translates to “humanness”, and refers to the behavior, moral attributes, upright values and attitudes where societal needs take precedence over individual needs and togetherness, sympathy, empathy and tolerance is valued. In addition, the Ubuntu philosophy is collectivist in approach, defined by the “educatedness” of a person and serves as a contradiction to an individualistic, Eurocentric approach to life and education. In an earlier study, Samkange and Samkange (1980) put forward three maxims of Ubuntu: Human relations where being human is found in recognizing the humanity of others; sanctity of life where preservation of life is more important than wealth; and people centered status where a leader's status is owed to the will of the people. Ubuntu is something that can be taught and mentored in schools and organizations. There needs to be an understanding of oneself as an individual as well as what one does as a professional (Teffo, 1999). Mugumbate and Chereni (2020, 6) concur with this view, and state Africans need to learn, write and practice Ubuntu.

Building on the perspectives of these highly regarded scholars, this study, however, aims to conceptualize *Ubuntu* leadership (in a positive, organizational sense) by exploring its drivers and outcomes, acknowledging the potential for negative aspects. For clarity, “*Ubuntu* leadership” in this context refers specifically to organizational settings, excluding broader cultural, political, or religious interpretations.

## 2 Materials and methods

Seminal scholars have established that Ubuntu and Ubuntu leadership are multi-faceted constructs, yet there is a lack of empirical qualitative explorative research supporting this claim. Although Molose et al. (2019) conducted an empirical study confirming the multi-dimensional nature of Ubuntu within a specific sector, the interactions between its components are often hypothesized or omitted in scholarly work. To address this, the study employs the Interactive Qualitative Analysis (IQA) methodology, which explores the interactions from drivers to outcomes of Ubuntu leadership. The purpose of IQA is to create a visual representation of the influences and outcomes of Ubuntu leadership, which is focused on the sub-elements of the construct, the inter-relations from a contextual and community-driven perspective. IQA allows participants (organizational leaders) to define Ubuntu leadership based on their lived experiences, providing an context specific perspective. Data collection involves facilitated group processes or focus groups (Bargate, 2014; Northcutt and McCoy, 2004), with participant selection based on their power over and knowledge of the phenomenon. This study focuses on South African organizational leaders, utilizing three purposive focus groups of 9 to 17 participants, totaling 36. Group one met face-to-face, while groups two and three met online via Zoom for practicality. Northcutt and McCoy (2004) recommend focus group membership of up to 12 participants, but it was decided to involve 17 participants due to the online nature of focus group 3. Data collection began with a silent brainstorming phase based on the issue statement regarding Lovemore Mbigi's work on Ubuntu. Participants reflected on how Ubuntu influences their leadership experiences by considering their surroundings, feelings, and behaviors as Ubuntu leaders. Participants recorded their thoughts on paper or via Zoom Group Chat, followed by a facilitated discussion to clarify meanings. The affinity analysis involved inductive and axial coding, where participants grouped similar responses to form affinities, which were then named and defined collaboratively. Each group identified affinities through physical or digital means, resulting in six affinities from group one and five from groups two and three. During axial coding, small groups defined affinities and reached consensus on their meanings. Theoretical coding followed, linking affinities to establish cause-and-effect relationships using Affinity Relationship Tables (ARTs). Each focus group produced several ARTs, which were combined into composite ARTs for subsequent analysis. The next step involved creating inter-relationship diagrams (IRDs) for each focus group from the composite ARTs, applying the Pareto principle to identify significant relationships. This led to the development of a System Influence Diagram (SID), visually representing the system of influences and outcomes. The SID illustrated the dynamics of Ubuntu leadership and highlighted potential areas for influence and change.

### 2.1 Rigor and choice of method

Both researchers recognize that their social, cultural, and personal identities influence their perspectives. To minimize subjectivity, IQA was selected as the research methodology due to its participatory, transparent, and reflexive framework, which effectively addresses positionality by reducing researcher bias and promoting cultural sensitivity. No significant power imbalances with participants were identified in this study; however, any potential imbalances were methodologically addressed through active listening, collaboration with the community, and transparent processes. The researchers are dedicated to ethical research practices that prioritize participants' voices, including securing culturally sensitive informed consent, ensuring anonymity, and sharing findings with the community for validation.

In summary, IQA reduces the influence of the researcher's cultural or personal biases by prioritizing participants' perspectives. The developers of the technique, Northcutt and McCoy (2004) and Bargate (2014), postulate that principles of IQA support credibility, transferability and dependability while highlighting the concepts of validity and reliability through accessible and transparent procedures. It was subsequently decided to use the IQA as method to investigate a phenomenon from an explorative, qualitative perspective, due to its contextual and community-driven nature, emphasizing collective meaning-making, relationships, and interconnectedness. It allows organizational leaders to define *Ubuntu* leadership from their lived experiences, making it a truly context sensitive conceptualization of the construct directly grounded in participants” cultural realities, the results have high contextual validity.

The IQA ensures involvement, participation, shared meaning-making, and the co-creation of knowledge. Participants analyse and interpret the data, and the researcher fulfills the role of facilitator, which minimizes any biases and prejudices. This ensures that the voice of the participants is valued. This allows for a holistic understanding of cultural dynamics as perceived by insiders (co-creators), which is crucial in context specific, explorative qualitative research. This approach differs from many of the traditional qualitative methods often involve heavy researcher interpretation, which can introduce etic (outsider) bias.

Consequently, the data generated from the IQA sessions in this study is directly grounded in the perspectives of the 36 organizational leaders from various races that took part. The sampling in this study was purposeful, designed to select participants who can provide meaningful insights into the phenomenon under study while supporting the methodology's participatory and reflexive nature. The participants were organizational leaders deeply embedded in the context of the study (organizational leadership) were able to articulate their lived experiences, ensuring that the data reflects their subjective realities. The characteristics of the participants are reported in Table 1.

**Table 1:** Characteristics of the participants.

**Grouping variable**		**Focus group** ^**1**^ ***N*** = **10**		**Focus group** ^**2**^ ***N*** = **9**		**Focus group** ^**3**^ ***N*** = **17**	
		***n***	**%**	**n**	**%**	**n**	**%**
**Sector**	Private	9	90	8	89	17	100
	Public	1	10	1	11		
**Gender**	Male	3	30	4	44	5	29
	Female	7	70	5	56	12	71
**Education**	Grade 12	3	30			6	35
	1^st^ degree/diploma	3	30	3	33	6	35
	Higher degree/diploma	2	20	4	45	4	24
	Master's degree	1	10	2	22	1	6
	Doctoral degree	1	10				
**Race**	African	2	20	5	56		
	Indian					9	53
	White	8	80	4	44	7	41
	Multiple race					1	6
**Years in leader-ship role**	< 10	6	60	4	44	7	41
	11–20	2	20	1	11	6	35
	20+	2	20	3	33	4	24
**Frequency of contact with other leaders**	Minimal	1	10	1	11		
	Daily	4	40	4	44	16	94
	Weekly	5	50	1	11	1	6
	Monthly			2	22		
**Mean age (years)**	40.00 (*SD* = 7.80)		39.00 (SD = 9.89)		44.12 (SD = 7.93)		
**Mean years in a leadership role (years)**	8.50 (SD = 8.46)		12.56 (SD = 8.84)		11.06 (SD = 7.57)		

Focus group 1 consisted of 10 participants spanning the automotive, financial planning, consulting and research and development industries. Seven participants were female and three were male. The racial composition of the group consisted of eight whites and two blacks. Their ages ranged from 28–50 years old (*M* = 40.00; *SD* = 7.80). The group's education level ranged from matric through to doctoral level and years in a leadership role ranged from 9 months to 25 years (*M* = 8.50; *SD* = 8.46).

Focus group 2 consisted of nine participants from the electrical and telecommunication design, construction, adult education, entrepreneurship development, agriculture (cannabis), ICT software development, timber (sawmilling), manufacturing (food and beverage) and automotive (learning and development) industries. Five participants were female and four were male with their ages ranging from 24–59 years old (*M* = 39.00; *SD* = 9.89). The racial composition of the group was five blacks and four whites. The group's education level ranged from BTech through to Master's degree and years in a leadership role ranged from one to 30 years (*M* = 12.56; *SD* = 8.84).

There were 17 participants in focus group three from private sector organizations, from industries that included insurance, financial services and administration. 12 participants were female and five were male with their ages ranging from 32–59 years old (*M* = 44.12; *SD* = 7.93). The group consisted of nine Indian, seven White participants and one participant from the multiple race category. The group's education level ranged from matric/Grade 12 to MBA, CA (SA) and LLB and years in a leadership role ranged from 10 months to 30 years (*M* = 11.06; *SD* = 7.57).

After inspection of Table 1, it is important to mention that the sample is comprised of a majority of cultural outsiders to the ubuntu (Nguni) lived experience but well versed in the realities of *Ubuntu* as an organizational leadership phenomenon, which is the focus of this study. These realities, as structured information, were clarified and coded in a facilitated process, limiting the researchers' interpretation, through a rationalized methodological process. This process ensures that knowledge is co-constructed from shared meanings, aligned with the constructivist emphasis on subjective, context-specific realities.

## 3 Results

The results of the affinity analysis of each of the three focus groups are discussed in this section.

### 3.1 Affinity analysis focus group 1

The affinity analysis conducted with Focus Group 1 identified six key thematic areas (affinities), representing the participants” collective understanding of *Ubuntu* leadership within organizational contexts. These affinities encapsulate both the aspirational qualities and practical challenges associated with applying *Ubuntu* principles in leadership. They are:

- *Outcome of Ubuntu leadership* emphasizes the ultimate goal of fostering a positive organizational culture rooted in shared values like compassion, cultural awareness, and productivity.
- *DNA of Ubuntu* captures the foundational elements of inclusivity, shared learning, and mutual understanding necessary to make *Ubuntu* viable in practice.
- *Unpredictable challenge* acknowledges the complexity and messiness of implementing *Ubuntu* leadership, including risks such as misinterpretation, lack of accountability, and the difficulty of achieving consensus.
- *Authentic integrity* calls for honesty, collaboration, and ongoing self-assessment, recognizing that true *Ubuntu* leadership requires individuals to act with integrity and transparency.
- *Ubuntu communication and honesty* highlights the critical role of open, honest communication in fostering harmony and achieving collective goals, with an emphasis on fairness, cooperation, and discipline.
- *Moving forward together* reflects the need for a balanced approach that values peace, personal growth, and collective responsibility, while remaining mindful of productivity in a business setting.

Together, these affinities portray *Ubuntu* leadership as a nuanced, value-driven approach that blends empathy and collaboration with the realities of organizational life.

### 3.2 Theoretical coding and system influence diagram focus group 1

The focus group 1 affinities were subjected to a theoretical coding process and Pareto Analysis to establish the direction of influence between them as per tabular IRD in Table 2.

**Table 2:** Tabular inter-relationship diagram in descending order – focus group 1.

**Tabular IRD in descending order**										**SID assignments**
**#**	**1**	**2**	**3**	**4**	**5**	**6**	**OUT**	**IN**	Δ	
5	↑		↑	↑		↑	4	0	4	*Ubuntu* communication and honesty (PRIMARY DRIVER)
3	↑	↑		↑		↑	4	1	3	The unpredictable challenge (SECONDARY DRIVER)
2	↑		←	↑		↑	3	1	2	DNA of *Ubuntu* (SECONDARY DRIVER)
4	↑	←	←		←	↑	2	3	−1	Authentic integrity (SECONDARY OUTCOME)
1		←	←	←	←	↑	1	4	−3	Outcome of *Ubuntu* leadership (SECONDARY OUTCOME)
6	←	←	←	←	←		0	5	−5	Moving forward together (PRIMARY OUTCOME)

*Where:* 1= Outcome of *Ubuntu* leadership; 2= DNA of *Ubuntu*; 3= Unpredictable challenge; 4= Authentic integrity; 5= *Ubuntu* communication and honesty, and 6= Moving forward together, *with* (↑) = Out; (←) = In, and Δ = Out- In.

The Influence Diagram (SID), organizing the six identified affinities into three drivers and outcomes respectively. The key drivers of *Ubuntu* leadership were identified as *Ubuntu communication and honesty* (primary driver), *Unpredictable challenge*, and the *DNA of Ubuntu* (secondary drivers). These reflect the importance of honest communication, embracing the complexities of *Ubuntu* leadership, and fostering a culture of inclusiveness and shared learning.

These foundational elements were seen to lead to three outcomes: *Authentic integrity*, the *Outcome of Ubuntu leadership* (as secondary outcomes), and *Moving forward together* as the primary outcome. The group concluded that effective *Ubuntu* leadership manifests through leaders who are authentic and act with integrity, cultivating shared goals grounded in compassion, cultural awareness, and productivity. Ultimately, this creates a supportive environment where team members can grow, collaborate effectively, and move forward collectively, contributing to a positive organizational culture and improved performance.

### 3.3 Affinity analysis focus group 2

Focus Group 2 identified five core affinities reflecting their shared understanding of *Ubuntu* leadership in the workplace. These affinities portray a leadership approach centered on inclusivity, empathy, and collaboration, driving organizational effectiveness and team success. They are:

- *Transformational agent* emphasizes the role of authentic leaders who value diversity, communicate inclusively, and inspire trust by promoting shared values and a unifying vision.
- *Organizational culture* highlights the creation of an inclusive, community-based environment that supports continual improvement and aligns individual, team, and organizational goals.
- *Joining forces* describes collaborative, participative processes that unite team members around common goals, encouraging mutual support, co-creation, and the celebration of achievements.
- *Empath*y is central to the *Ubuntu* leadership philosophy, focusing on compassionate communication, trust-building, and support for employee wellbeing.
- Team performance is the primary outcome of this leadership model, driven by self-leadership, accountability, innovation, and collective responsibility—leading to higher engagement and organizational success.

Collectively, these affinities illustrate how *Ubuntu* leadership integrates human-centered values with practical strategies to build strong, high-performing teams.

### 3.4 Theoretical coding and system influence diagram focus group 2

The affinities were subjected to a theoretical coding process and Pareto Analysis to establish the direction of influence between the affinities as per tabular IRD in Table 3.

**Table 3:** Tabular inter-relationship diagram in descending order – focus group 2.

**Tabular IRD in descending order**									**SID assignments**
**#**	**1**	**2**	**3**	**4**	**5**	**OUT**	**IN**	Δ	
1		↑		↑	↑	3	0	3	Transformational Agent (PRIMARY DRIVER)
4	←	↑	↑		↑	3	1	2	Empathy (SECONDARY DRIVER)
3				←	↑	1	1	0	Joining Forces (PIVOT)
2	←			←	↑	1	2	−1	Organizational Culture (SECONDARY OUTCOME)
5	←	←	←	←		0	4	−4	Team Performance (PRIMARY OUTCOME)

*Where:* 1= Transformational Agent; 2= Organizational Culture; 3= Joining Forces; 4= Empathy, and 5= Team Performance, *with* (↑) = Out; (←) = In, and Δ = Out- In.

Focus group 2′s affinity analysis resulted in five key themes (affinities) that define the characteristics and outcomes of *Ubuntu* leadership in an organizational context. These were structured into a system influence diagram (SID), identifying two drivers, one pivot, and two outcomes.

The drivers were *Transformational agent* and *Empathy* as primary and secondary drivers respectively. This highlights the importance of leaders who are authentic, value diversity, communicate inclusively, and lead with compassion. These leaders are seen as central to initiating and sustaining *Ubuntu* leadership by fostering trust, unity of purpose, and emotional intelligence.

The pivot is *Joining forces* and represents the collaborative and participative processes that unite individual strengths into cohesive teams. This element is essential in translating leadership values into action through co-creation, shared goals, and collective motivation.

The outcomes were identified as *Organizational culture* (secondary outcome) and *Team performance* (primary outcome). The resulting culture is inclusive, community-based, and focused on continuous improvement and mentorship. The ultimate outcome, *Team performance*, is characterized by self-leadership, accountability, critical thinking, and innovation—reflecting the mature and empowered teams that *Ubuntu* leadership aims to build.

Together, the system outlines a clear flow from empathetic, transformational leadership to strong team performance through collaboration and inclusive culture-building.

### 3.5 Affinity analysis focus group 3

Focus Group 3 identified five key affinities that reflect their collective understanding of *Ubuntu* leadership as a people-centered, inclusive, and growth-oriented approach. The themes emphasize the development of individuals and teams through empathy, shared knowledge, and mutual respect. They are:

- *Transformational agent* emphasizes the role of authentic leaders who value diversity, communicate inclusively, and inspire trust by promoting shared values and a unifying vision.
- *Nurturing through knowledge* highlights the importance of continuous personal and collective growth, where sharing knowledge and empowering others leads to more fulfilled, productive individuals who contribute positively to society.
- *Stronger people make people stronger* focuses on compassion, trust, and understanding as foundations for building strong character and inclusive leadership. This affinity stresses the value of empathy, clear communication, and authenticity in developing confident and capable individuals.
- *Inclusive team–driving success through others* underlines the power of collaborative and supportive teams. By fostering open communication and putting the team first, leaders create harmony and enable higher performance through shared success.
- *The front line* represents the steady and organized leadership qualities of high-performing individuals who bring consistency, calmness, and structure to their teams, leading to a harmonious and efficient work environment.
- *Simunye – We are one* encapsulates the spirit of unity and mutual respect. It stresses active listening, cultural awareness, and creating safe spaces where every team member's voice is valued, helping to build trust and inclusivity.

Together, these affinities portray *Ubuntu* leadership as a holistic, empathetic, and empowering practice that fosters strong individuals, cohesive teams, and a respectful, high-functioning organizational culture.

### 3.6 Theoretical coding and system influence diagram focus group 3

The affinities were subjected to a theoretical coding process and Pareto Analysis to establish the direction of influence between the affinities as per tabular IRD in Table 4.

**Table 4:** Tabular inter-relationship diagram and SID of focus group 3.

**Tabular IRD in descending order**									**SID assignments**
**#**	**1**	**2**	**3**	**4**	**5**	**OUT**	**IN**	Δ	
1		↑	↑	↑	↑	4	0	4	Nurturing through knowledge (PRIMARY DRIVER)
2	←		↑	↑		2	1	1	Stronger people make other people stronger (SECONDARY DRIVER)
5	←		↑	↑		2	1	1	Simunye (We are one) (SECONDARY DRIVER)
3	←	←		↑	←	1	3	−2	Inclusive team: Driving success through others (SECONDARY OUTCOME)
4	←	←	←		←	0	4	−4	The front line (PRIMARY OUTCOME)

*Where*: 1. Nurturing through knowledge 2. Strong people make other people stronger 3. Inclusive team: Driving success through others 4. The front line 5. Simunye (*we are one*), *with* (↑) = Out; (←) = In, and Δ = Out- In.

The outcome of focus group 3 is reported in Table 4. The three drivers are *Nurturing through knowledge* (primary driver) and *Stronger people make other people stronger* as well as *Simunye* – *we are one* as secondary drivers, respectively. Consequently, focus group 3 felt that sharing knowledge to promote ongoing growth and development of individuals, being compassionate and understanding, listening actively, creating a safe space for the contribution of ideas as well as earning respect will drive organizational *Ubuntu* leadership. The two outcomes in the system are *Inclusive team: driving success through others* as secondary outcome and *the front line* as primary driver, associated with flexible, supportive and collaborative teams that are structured and well organized. In addition, the leaders are consistent, calm, considered, orderly, efficient and harmonious, thus providing a peaceful working environment.

## 4 Discussion

The first affinity group included the drivers across the systems: *Ubuntu* communication and honesty (focus group 1), transformational agent (focus group 2), empathy (focus group 2), nurturing through knowledge (focus group 3) and stronger people make other people stronger (focus group 3). These affinities highlighted the importance of having a clear vision; the strength of diversity; effective, honest and empathetic communication; sharing of knowledge and skills to empower; and the importance of listening, empathy and shared values. This affinity group is supported in literature (for example Mangaliso, 2001; Mangaroo-Pillay, 2025; Nelwamondo and Price, 2025; Zondo, 2022) and is collectively named *Transformational focus and ability*.

The second affinity group included secondary drivers from focus group 1 only: unpredictable challenge and DNA of *Ubuntu*. These affinities highlighted the fact that it is not easy being an *Ubuntu* leader within an organizational context and that it is important to understand *Ubuntu*, to understand each other, to be accountable to one another and avoid unhealthy co-dependencies. This affinity group is supported in literature (Grobler and Koen, 2024; Mangaliso et al., 2021; Mangaroo-Pillay, 2025; Nelwamondo and Price, 2025; Nicolaides and Duho, 2019; Sipondo, 2025; Van Norren and Beehner, 2021), and is named *Authenticity, ethics and accountability*.

The third affinity group included the pivot affinity from focus group 2, joining forces, and a secondary outcome affinity from focus group 3, inclusive team–driving success through others. These affinities highlighted participative processes, togetherness, collaboration and common goals. This affinity group is supported in one of the themes identified in the systematic literature review conducted by authors prior to this study (*Ubuntu*-related leadership can be described as participatory and values based) as well as by Grobler and Singh (2018) and Mangaliso et al. (2021). The pivot in the intuitively created SID is called *Team synergy*.

The fourth affinity group consisted of three (3) affinities across all three systems. Authentic integrity, a secondary outcome from focus group 1; organizational culture, a secondary outcome from focus group 2; and *Simunye*–we are one, a secondary driver from focus group 3. These affinities highlighted the importance of being real and assessing a situation continually, as well as of a community-based sense of belonging and team-based organizational culture held together by dignity, trust and respect. This affinity grouping aligns with the isiXhosa proverb, “*umntu ngumntu ngabantu*” (Mangaliso, 2001, p. 24), which means a person is a person through others as well as Mbigi's (2007) five social values of survival, solidarity, compassion, respect and dignity. It is collectively named *Harmonious inclusive leadership and culture*.

The fifth affinity group included the primary outcomes across all three systems as well as a secondary outcome from focus group 1. These affinities were the following: outcome of *Ubuntu* leadership (focus group 1), moving forward together (focus group 1), team performance (focus group 2) and the front line (focus group 3). These affinities spoke to the presence or evidence of *Ubuntu* leadership, i.e. common goals and momentum; an enabling environment where individuals and teams can grow; and calm, consistent, peaceful, accountable and efficient self-leadership. This in essence describes the emotive, positive and promising future that the organizational leaders desire as a result of an *Ubuntu* leadership approach, and is collectively named *Individual, team and organizational growth and success*, and is supported by *Ubuntu* related literature (Mangaliso et al., 2021; Nelwamondo and Price, 2025; Osa, 2019).

**Figure 1:**
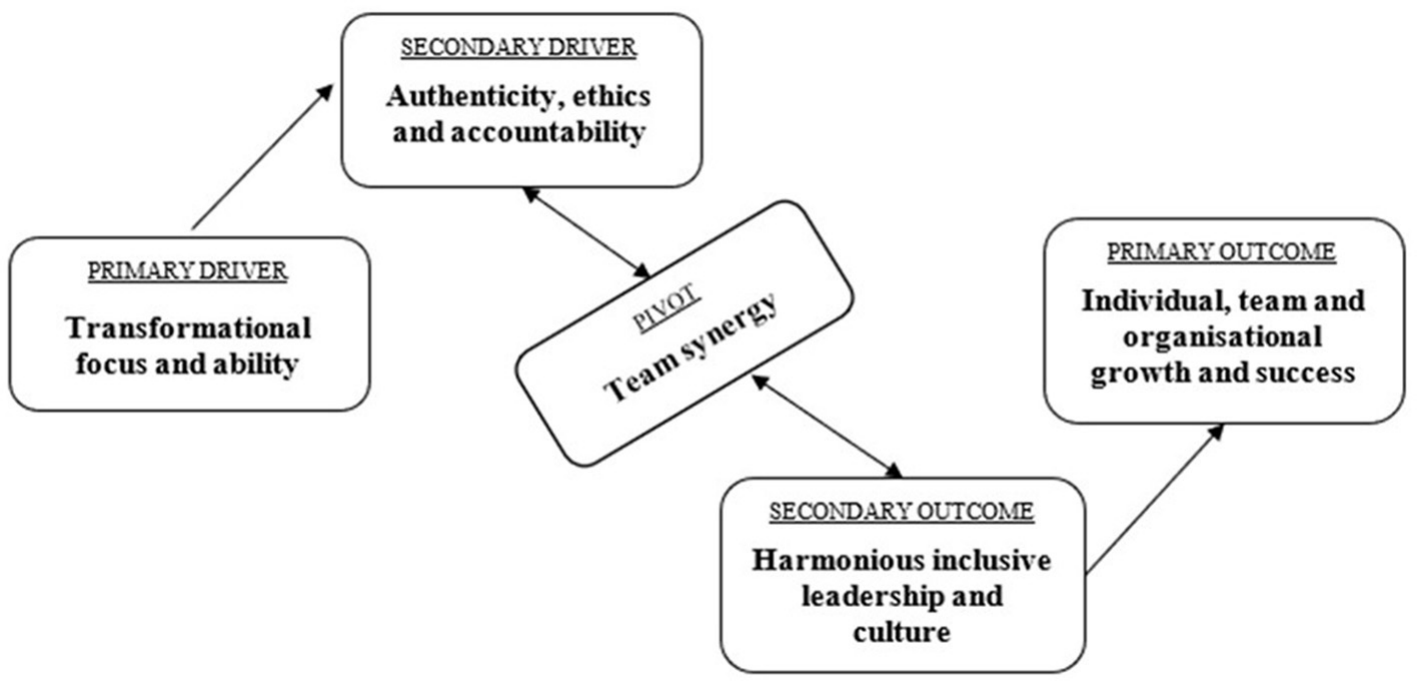
Combined uncluttered system influence diagram across all three (3) focus groups.

The findings of this study are depicted in the combined system influence diagram depicted in Figure 1, and summarized in Table 5.

**Table 5:** Summary of the findings.

**Collective naming**	**Affinity**	**Description**
**Transformational focus and ability** (PRIMARY DRIVER)	*Ubuntu* communication and honesty (focus group 1) transformational agent (focus group 2), nurturing through knowledge (focus group 3)	Having a clear vision; the strength of diversity; effective, honest and empathetic communication; sharing of knowledge and skills to empower; and the importance of listening, empathy and shared values.
**Authenticity, ethics and accountability** (SECONDARY DRIVER)	Authentic integrity (focus group 1), Simunye – *we are one* (focus group 3) Unpredictable challenge and DNA of *Ubuntu* (focus group 1), empathy (focus group 2); stronger people make other people stronger (focus group 3)	Trusted leaders, respecting everyone, honest, loyal and good judgement. Challenges of being an *Ubuntu* leader within an organizational context and that it is important to understand *Ubuntu*, to understand each other, to be accountable to one another and avoid unhealthy co-dependencies.
**Team synergy** (PIVOT)	Joining forces (focus group 2)	Value participative processes in which individual strengths are joined to create collaborative teams motivated through their participation in a supportive team environment, in which they work toward a common goal and reflect on and celebrate small wins.
**Harmonious inclusive leadership and culture** (SECONDARY OUTCOME)	Outcome of *Ubuntu* leadership (focus group 1), organizational culture (focus group 2) and an inclusive team – driving success through others (focus group 3)	Togetherness, harmony, common goal and momentum, positive, nurturing. The organizational culture supports our values of transformation through diverse and inclusive mindsets and behaviors to drive a community-based sense of belonging. Continual improvement and a mentorship culture drives efficiencies across the organization, incorporating both personal, team and organizational objectives. Teams are flexible, supportive and collaborative, allowing people to be comfortable and express themselves thereby achieving more.
**Individual, team and organizational growth and success** (PRIMARY OUTCOME)	Moving forward together (focus group 1), group performance (focus group 2) and the front line (focus group 3)	Common goals and momentum; an enabling environment where individuals and teams can grow; and calm, consistent, peaceful, accountable and efficient self-leadership. This in essence describes the emotive, positive and promising future that the organizational leaders desire due to the practicing of *Ubuntu* leadership.

Intuitively, the affinities were grouped across the three focus groups, with the combined results of the IQA process depicted in the system influence diagram below.

### 4.1 Limitations of the study

This study explicitly acknowledges its limited exploration of the fundamental epistemological contestation between *Ubuntu* and leadership in their purest forms, recognizing this as a high-level limitation. This study attempted to blend the two concepts (as lived experiences), and although it has value on a practical organizational leadership level, it lacks authentic philosophical integration. Future studies should rigorously and deliberately examine the underlying assumptions embedded in each paradigm.

The study further acknowledges certain methodological limitations, notably the sample's limited insider perspective on *Ubuntu* as a broader cultural phenomenon. While the cross-sectional design (three independent focus groups) provides insights into organizational *Ubuntu* leadership, it falls short of capturing its authentic cultural essence. Future research should adopt an insider, culturally grounded approach to link organizational practices with traditional roots, using multistage sampling to include deeply immersed participants for a more representative, contextually rich sample.

## 5 Conclusion and recommendations

With these limitations in mind, consistent findings across the three groups frame *Ubuntu* leadership as a transformative, inclusive, and ethical model that prioritizes people and relationships. Although each group offered a distinct perspective, namely managing complexity (Group 1), fostering empathy and cultural change (Group 2), and promoting personal and team development (Group 3), they collectively portray *Ubuntu* as a leadership philosophy that harmonizes humanity with performance, thus integrating *Ubuntu* with leadership in its general (Westernized) form.

The similarities that emerge across the affinity groups and *Ubuntu* leadership literature in an organizational context further include compassion for each other; treating each other with dignity and respect; demonstrating values-based behaviors; being relational; and being responsive toward one another, i.e., participatory and togetherness. The IQA process has confirmed and offered new insights into *Ubuntu* in an organizational leadership context through the perspectives of organizational leaders. The contribution is mainly in terms of the elements of *Ubuntu* identified and its inter-relational nature, from drivers, pivots and outcomes. This provides a unique view of the phenomena which has mainly been studied from a philosophical and at best, and etic approach.

This study offers a context sensitive conceptualization of *Ubuntu* leadership, which can be used by organizations, educators and scholars to promote *Ubuntu* leadership in practice, through leadership training and development interventions, the development of measurement instruments and the determination of *Ubuntu*'s effect on various organizational variables, such as organizational behavior, performance, etc. Leadership development programs must proactively mitigate the risk of epistemic confusion by preventing (i) the reduction of Ubuntu to a superficial corporate slogan and (ii) the cosmetic softening of Western leadership without substantive structural reform.

## Data Availability

The original contributions presented in the study are included in the article/supplementary material, further inquiries can be directed to the corresponding author.
